# Cardioprotective Effects of 1,3 Butanediol in MASLD via Reversal of Cardiac Lipid Accumulation and Suppression of Cardiac Fibrosis

**DOI:** 10.3390/ijms27125354

**Published:** 2026-06-13

**Authors:** Olufunto O. Badmus, Landon D. Parrow, Karis E. McGowen, LaBrenda Bell, Jennifer R. Greer, Marcela de Carvalho Cruz, Terry D. Hinds, David E. Stec

**Affiliations:** 1 Department of Physiology & Biophysics, Cardiovascular-Renal Research Center, Cardiorenal, and Metabolic Diseases Research Center, University of Mississippi Medical Center, Jackson, MS 39216, USA; obadmus@umc.edu (O.O.B.); ldp225@msstate.edu (L.D.P.); karimack997@gmail.com (K.E.M.); sidbell2006@gmail.com (L.B.); jennifer.greer722@gmail.com (J.R.G.); marcelacarvalhocruz@hotmail.com (M.d.C.C.); 2 Drug & Disease Discovery D3 Research Center, Department of Pharmacology and Nutritional Sciences, University of Kentucky College of Medicine, Lexington, KY 40536, USA; terry.hinds@uky.edu

**Keywords:** peroxisome proliferator-activated receptor α, hypertension, fatty liver

## Abstract

Metabolic dysfunction-associated steatotic liver disease (MASLD) is highly associated with the development of cardiovascular disease (CVD); however, the mechanisms responsible are currently unknown. We have developed a model of MASLD due to the loss of hepatocyte peroxisome proliferator-activated receptor α (PPARα^HEPKO^). We found that plasma beta-hydroxybutyrate (BHOB) levels were significantly reduced in PPARα^HEPKO^ mice and aimed to investigate the therapeutic potential of restoring BHOB levels in the development of CVD in these mice. Thirty-week-old PPARα^HEPKO^ and control PPARα^FL/FL^ mice were randomized to receive 1,3 butanediol (1,3-BDO), a precursor of BHOB, in drinking water for 6 weeks. 1,3-BDO treatment resulted in a significant increase in plasma BHOB levels, a significant decrease in mean arterial blood pressure, improvement in systolic and diastolic function, a decrease in vascular stiffness, and improved exercise performance in PPARα^HEPKO^ mice. 1,3-BDO treatment did not alleviate hepatic steatosis in PPARα^HEPKO^ mice; however, it improved plasma cholesterol levels and decreased cardiac lipid accumulation, fibrosis, and apoptosis. 1,3-BDO treatment also resulted in a significant increase in cardiac AMP-activated protein kinase (AMPK) levels. Increasing plasma BHOB levels reverses CVD in our mouse model of MASLD. A similar approach could be an effective strategy for preventing the development of CVD in patients with human MASLD.

## 1. Introduction

Metabolic dysfunction-associated steatotic liver disease (MASLD) is a multifactorial disease in which the liver accumulates excessive lipids in the presence of metabolic diseases such as obesity and diabetes. While the vast majority of patients who have this disease are obese or diabetic, there is a subgroup of patients who exhibit excessive hepatic lipid accumulation without overt traditional metabolic risk factors [1,2]. These patients are referred to as having “lean” MASLD, although there is evidence that these patients have greater central obesity as opposed to an increased BMI [3,4]. The leading cause of mortality in patients with MASLD is cardiovascular disease (CVD), irrespective of whether the patient exhibits “lean” MASLD or not [5,6,7,8]. There is no single specific mechanism for CVD development in MASLD; it likely arises from several potential mechanisms, including hypertension, increased vascular stiffness, and alterations in hepatokines and liver-derived metabolites.

We have recently described hepatocyte-specific peroxisome proliferator-activated receptor alpha (*Ppara^HEPKO^*) as a model of ‘lean’ MASLD [9]. These mice accumulate lipids in the liver, even on a standard laboratory diet, due to the loss of peroxisome proliferator-activated receptor α (PPARα), independent of changes in total body weight or the development of type II diabetes [9]. *Ppara^HEPKO^* mice also exhibit decreases in cardiac systolic and diastolic function, hypertension, and increased vascular stiffness, indicative of the development of CVD as seen in human patients. One of the strengths of this model is the opportunity to identify novel mechanisms that may link MASLD to the development of CVD. In this regard, *Ppara^HEPKO^* mice exhibit an interesting phenotype, characterized by a significant decrease in circulating levels of Beta-hydroxybutyrate (BHOB) [9]. Circulating levels of BHOB are derived from hepatic ketone metabolism, which is highly regulated by PPARα [10,11]. Several clinical studies have demonstrated a positive effect of ketogenic diets on the development of CVD [12,13]. Previous studies have shown that restoring plasma BHOB levels attenuates the development of hypertension in Dahl salt-sensitive rats [14]. The goal of the present study was to determine whether restoration of plasma BHOB levels confers cardioprotection against the development of CVD in *Ppara^HEPKO^* mice.

## 2. Results

### 2.1. Increasing Plasma BHOB Reduces Body and Adipose Tissue Weights in Ppara^FL/FL^ and Ppara^HEPKO^ Mice

**Figure 1:**
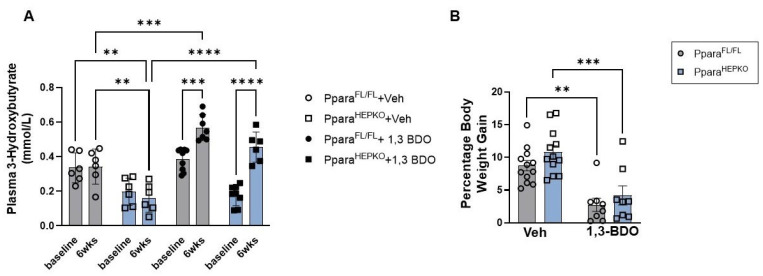
1,3-BDO treatment increased plasma BHOB and decreased body weight gain in the *Ppara^HEPKO^* mice. (**A**) Plasma BHOB, (**B**) body weight gain. Values are expressed as means ± SEM; ** = *p* < 0.01, *** = *p* < 0.001, **** = *p* < 0.0001; (**A**): *n* = 6 and 9 *Ppara^FL/FL^* VEH, 1,3-BDO; *n* = 6 mice *Ppara^HEPKO^* VEH, 1,3-BDO; (**B**): *n =* 12 and 8 mice *Ppara^FL/FL^* VEH, 1,3-BDO; *n* = 12 and 8 mice *Ppara^HEPKO^* VEH, 1,3-BDO. VEH, vehicle; 1,3-BDO, 1,3-Butanediol.

*Ppara^HEPKO^* mice had reduced plasma BHOB compared to *Ppara^FL/FL^* mice at baseline, and treatment with 1,3-BDO increased plasma BHOB levels in both *Ppara^FL/FL^* and *Ppara^HEPKO^* mice (Figure 1A). Importantly, 1,3-BDO increased plasma BHOB levels in *Ppara^HEPKO^* mice to those observed in *Ppara^FL/Fl^* mice (Figure 1A). Body weight and body weight gain between *Ppara^FL/FL^* and *Ppara^HEPKO^* mice given vehicle were comparable; however, at 6 weeks of 1,3-BDO treatment, body weights and body weight gain were significantly reduced in both *Ppara^FL/FL^* and *Ppara^HEPKO^* mice (Table 1, Figure 1B). Heart and liver weights normalized to body weight were not significantly different between the groups, but kidney weight, when normalized to body weight, was significantly higher in the *Ppara^HEPKO^* mice treated with 1,3-BDO (Table 1). Next, we examined the effect of 1,3-BDO treatment on adipose depot distribution. *Ppara^HEPKO^* mice exhibited an increase in both epididymal and visceral fat mass as compared to *Ppara^FL/FL^* mice which was reduced by 1,3-BDO treatment (Table 1). There were no changes in retroperitoneal fat pad depots between *Ppara^HEPKO^* and *Ppara^FL/FL^* mice, irrespective of treatment (Table 1).

### 2.2. 1,3-BDO Treatment Does Not Reduce Hepatic Lipid Accumulation but Improves Plasma Cholesterol in Ppara^HEPKO^ Mice

**Figure 2:**
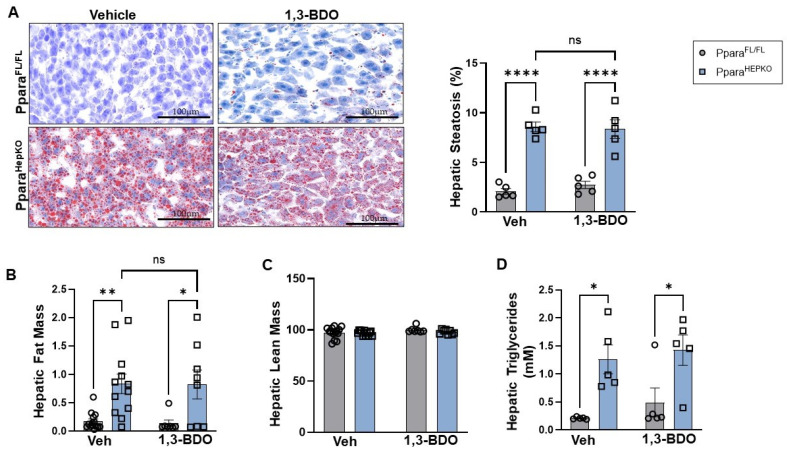
1,3-BDO treatment does not attenuate hepatic lipid accumulation in *Ppara^HEPKO^* mice. (**A**) Representative Oil Red O staining of liver tissue. (**B**) Hepatic fat mass. (**C**) Hepatic lean mass. (**D**) Hepatic triglyceride levels. Values are expressed as means ± SEM; * = *p* < 0.05, ** = *p* < 0.01, **** = *p* < 0.0001. (**A**): *n* = 5/group; (**B**): *n =* 14 and 7 mice *Ppara^FL/FL^* VEH, 1,3-BDO; *n* = 12 and 8 mice *Ppara^HEPKO^* VEH, 1,3-BDO; (**C**): *n =* 14 and 7 mice *Ppara^FL/FL^* VEH, 1,3-BDO; *n* = 12 and 8 mice *Ppara^HEPKO^* VEH, 1,3-BDO. Scale bar = 100 μm. Statistical analyses were performed using two-way ANOVA with genotype and treatment as factors, followed by Tukey post hoc test for all subsequent pair-wise comparisons. ns = not significant.

Hepatic fat accumulation, as measured by Oil Red O staining of liver sections, demonstrated significantly higher lipid accumulation in *Ppara^HEPKO^* mice compared to *Ppara^FL/FL^* mice. Interestingly, 1,3-BDO treatment of *Ppara^HEPKO^* mice did not lower hepatic lipid levels as compared to vehicle-treated *Ppara^HEPKO^* mice (Figure 2A). Hepatic lipid accumulation was further determined by measuring hepatic fat and lean mass with EchoMRI. The results from the EchoMRI mirrored those of Oil Red O, as no significant differences in hepatic fat levels were observed between vehicle- and 1,3-BDO-treated *Ppara^HEPKO^* mice (Figure 2B,C). Lastly, we confirmed these measurements with direct measurement of hepatic triglycerides. Hepatic triglyceride levels were not significantly different between vehicle- or 1,3-BDO-treated *Ppara^HEPKO^* mice (Figure 2D). Overall, these data demonstrate that at the concentration used in the present study, 1,3-BDO treatment does not decrease hepatic lipid accumulation in *Ppara^HEPKO^* mice maintained on a standard laboratory diet. Despite not having any effect on hepatic lipid accumulation, 1,3-BDO treatment had a significant effect on lowering plasma cholesterol, LDL cholesterol, AopA1, and cholesterol particles in *Ppara^HEPKO^* mice (Table 2). 1,3-BDO treatment also decreased plasma citric acid and ethanol levels in *Ppara^HEPKO^* mice (Table 2).

### 2.3. 1,3-BDO Treatment Lowers Blood Pressure and Attenuates Vascular Stiffness in Ppara^HEPKO^ Mice

**Figure 3:**
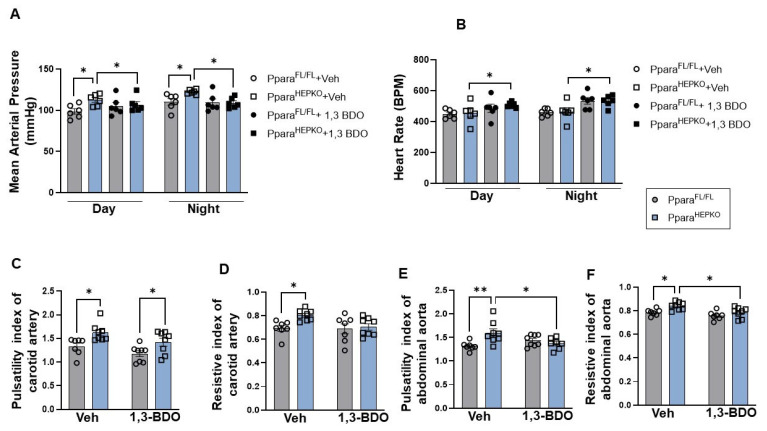
1,3-BDO treatment lowered blood pressure and decreased vascular stiffness in the *Ppara^HEPKO^* mice. (**A**) Day and night mean arterial pressure (MAP) over the 7-day recording period, (**B**) day and night heart rate over the 7-day recording period, (**C**) pulsatility index of left carotid artery, (**D**) resistive index of left carotid artery, (**E**) pulsatility index of abdominal aorta, (**F**) resistive index of abdominal aorta. Values are expressed as means ± SEM; * = *p* < 0.05, ** *p* < 0.01, (**A**,**B**): *n* = 5/group; (**C**): *n =* 7 mice *Ppara^FL/FL^* VEH, 1,3-BDO; *n* = 8 mice *Ppara^HEPKO^* VEH, 1,3-BDO; (**D**): *n =* 7 mice *Ppara^FL/FL^* VEH, 1,3-BDO; *n* = 8 mice *Ppara^HEPKO^* VEH, 1,3-BDO; (**E**): *n =* 7 and 8 mice *Ppara^FL/FL^* VEH, 1,3-BDO; *n* = 8 and 7 mice *Ppara^HEPKO^* VEH, 1,3-BDO; (**F**): *n =* 7 mice *Ppara^FL/FL^* VEH, 1,3-BDO; *n* = 8 mice *Ppara^HEPKO^* VEH, 1,3-BDO; VEH, vehicle; 1,3-BDO, 1,3-Butanediol. Statistical analyses were performed using two-way ANOVA with genotype and treatment as factors, followed by Tukey post hoc test for all subsequent pair-wise comparisons.

Blood pressure was continuously recorded for 7 days via radiotelemetery in vehicle- and 1,3-BDO-treated *Ppara^HEPKO^* and *Ppara^FL/FL^* mice. Mean arterial blood pressure (MAP) was significantly higher in vehicle-treated *Ppara^HEPKO^* as compared to *Ppara^FL/FL^* mice (Figure 3A). 1,3-BDO treatment significantly lowered MAP in Ppara^HepKO^ mice but had no significant effect on blood pressure in *Ppara^FL/FL^* mice (Figure 3A). Vascular stiffness was assessed by measuring the pulsatility index (PI) and resistive index (RI) of the left carotid artery and the abdominal aorta. The PI and RI of the left carotid artery were significantly higher in *Ppara^HEPKO^* mice compared to Ppara^fl/fl^ mice, indicating vascular stiffness, which was reduced significantly by 1,3-BDO treatment (Figure 3C,D). Similarly, the increase in PI and RI of the abdominal aorta in *Ppara^HEPKO^* mice was significantly lowered by 1,3-BDO treatment in the *Ppara^HEPKO^* (Figure 3E,F). 1,3-BDO treatment had no significant effect on PI or RI in the carotid artery or the aorta in *Ppara^FL/FL^* mice (Figure 3C–F).

### 2.4. 1,3-BDO Treatment Improves Cardiac Systolic and Diastolic Function but Not Left Ventricular Remodeling in the Ppara^HEPKO^ Mice

**Figure 4:**
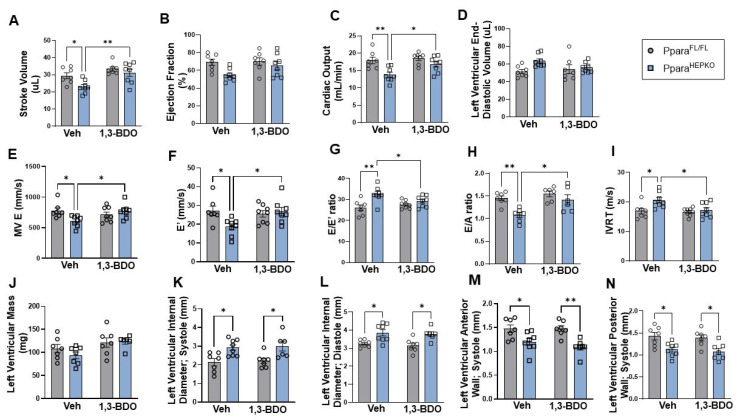
1,3-BDO treatment reversed systolic and diastolic dysfunction, but not cardiac remodeling, in *Ppara^HEPKO^* mice. (**A**) Stroke volume, (**B**) ejection fraction, (**C**) cardiac output, (**D**) left-ventricular end-diastolic volume, (**E**) peak early diastolic transmitral flow velocity (**E**), (**F**) mitral annular e’ velocity (e’), (**G**) ratio of E/e’, (**H**) ratio of E and late diastolic transmitral flow velocity (E/A), (**I**) isovolumic relaxation time (IVRT), (**J**) left ventricular mass, (**K**) left ventricular (LV) internal diameter during systole, (**L**) LV internal diameter during diastole, (**M**) LV anterior wall during systole, (**N**) LV posterior wall during systole. Values are expressed as means ± SEM; * = *p* < 0.05, ** *p* < 0.01; (**A**–**E**): *n =* 7 mice *Ppara^FL/FL^* VEH, 1,3-BDO; *n* = 8 mice *Ppara^HEPKO^* VEH, 1,3-BDO; (**F**,**G**): *n =* 7 and 8 mice *Ppara^FL/FL^* VEH, 1,3-BDO; *n* = 8 mice *Ppara^HEPKO^* VEH, 1,3-BDO; (**H**): *n =* 7 and 6 mice *Ppara^FL/FL^* VEH, 1,3-BDO; *n* = 7 and 6 mice *Ppara^HEPKO^* VEH, 1,3-BDO; (**I**): *n =* 7 and 8 mice *Ppara^FL/FL^* VEH, 1,3-BDO; *n* = 8 mice *Ppara^HEPKO^* VEH, 1,3-BDO; VEH, vehicle; 1,3-BDO, 1,3-Butanediol. Statistical analyses were performed using two-way ANOVA with genotype and treatment as factors, followed by Tukey post hoc test for all subsequent pair-wise comparisons.

Next, we determined the effect of 1,3-BDO treatment on cardiac function in *Ppara^HEPKO^* mice. We observed that 6 weeks of 1,3-BDO treatment reversed systolic dysfunction in *Ppara^HEPKO^* mice by increasing stroke volume (Figure 4A), ejection fraction (Figure 4B), and cardiac output (Figure 4C). Furthermore, 6 weeks of 1,3-BDO treatment improved diastolic function in *Ppara^HEPKO^* mice by restoring the peak wave velocity of blood flow across the mitral valve during early diastole (MV E) (Figure 4E) and reversing the reduced early diastolic mitral annular velocity (e’), which is a key indicator of diastolic function (Figure 4F). The elevated E/e’ ratio was also attenuated by 1,3-BDO treatment in *Ppara^HEPKO^* mice (Figure 4G). The ratio of the early to late diastolic transmitral flow velocities (E/A) was reduced in *Ppara^HEPKO^* mice as compared to *Ppara^FL/FL^* mice, and 1,3-BDO treatment restored this E/A ratio in Ppara^HepKO^ mice (Figure 4H). Another vital parameter that is used to determine LV diastolic function is isovolumic relaxation time (IVRT). We observed prolonged IVRT in *Ppara^HEPKO^* mice compared to *Ppara^FL/FL^* mice, which was reversed by 1,3-BDO treatment (Figure 4I). Data from our earlier study revealed cardiac remodeling that was characterized by LV dilation and thinness of the LV walls in *Ppara^HEPKO^* mice. Interestingly, in the present study, we observed that 1,3-BDO treatment had no effect on LV mass and remodeling in *Ppara^HEPKO^* mice (Figure 4J–N).

### 2.5. 1,3-BDO Improves Exercise Performance and Aerobic Capacity in Ppara^HEPKO^ Mice

**Figure 5:**
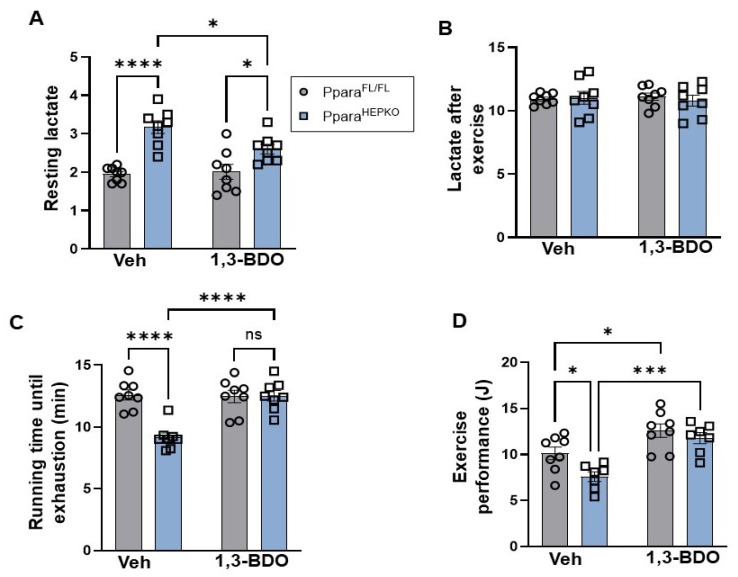
1,3-BDO treatment improves exercise tolerance in *Ppara^HEPKO^* mice. (**A**) Lactate at rest, (**B**) lactate after exercise, (**C**) running time until exhaustion, (**D**) exercise performance. Values are expressed as means ± SEM; * = *p* < 0.05, *** = *p* < 0.01, **** *p* < 0.0001; (**A**–**D**): *n =* 8 mice *Ppara^FL/FL^* VEH, 1,3-BDO; *n* = 8 and 7 mice *Ppara^HEPKO^* VEH, 1,3-BDO; VEH, vehicle; 1,3-BDO, 1,3-Butanediol. Statistical analyses were performed using two-way ANOVA with genotype and treatment as factors, followed by Tukey post hoc test for all subsequent pair-wise comparisons. ns = not significant.

We determined the effect of 1,3-BDO treatment on cardiovascular fitness via an exercise tolerance test. *Ppara^HEPKO^* mice exhibited increased resting lactate as compared to *Ppara^FL/FL^* mice, which was reduced with 1,3-BDO treatment (Figure 5A). 1,3-BDO treatment did not affect post-exercise blood lactate levels in either *Ppara^HEPKO^* or *Ppara^FL/FL^* mice (Figure 5B). Both running time until exhaustion (Figure 5C) and exercise performance (Figure 5D) were significantly improved by 1,3-BDO treatment in *Ppara^HEPKO^* mice.

### 2.6. 1,3-BDO Treatment Reverses Cardiac Lipid Accumulation and Fibrosis in the Ppara^HEPKO^ Mice

**Figure 6:**
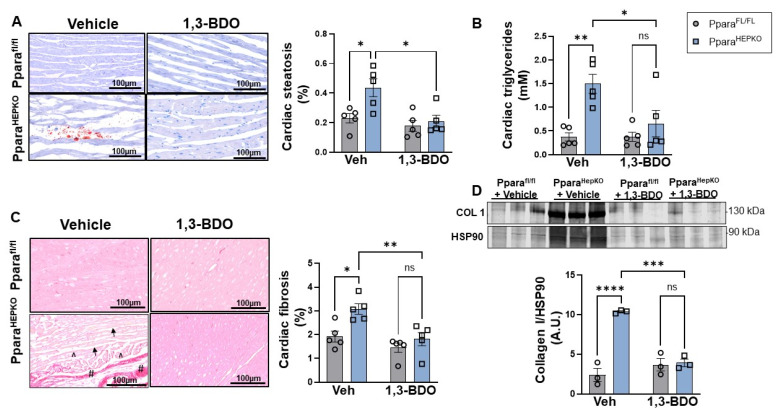
1,3-BDO treatment reverses cardiac lipid accumulation and collagen deposition in *Ppara^HEPKO^* mice. (**A**) Representative image of Oil Red O staining of cardiac tissue. (**B**) Cardiac triglyceride levels. (**C**) Representative image of picrosirius red staining of cardiac tissue. (**D**) Western blot of collagen I. Values are expressed as means ± SEM; * = *p* < 0.05, ** = *p* < 0.01, *** = *p* < 0.001, **** = *p* < 0.0001. (**A**): *n =* 5/group; (**B**,**C**): *n =* 3/group; VEH, vehicle; 1,3-BDO, 1,3-Butanediol. Scale bar = 100 μm. Statistical analyses were performed using two-way ANOVA with genotype and treatment as factors, followed by Tukey post hoc test for all subsequent pair-wise comparisons. Arrows indicate widened interstitial spaces between myocardial bundles. ^ indicates areas of fiber fractioning and spacing. # indicates collagen fiber deposition. ns = not significant.

Cardiac lipid accumulation, as assessed by Oil Red O staining, was significantly reduced by 1,3-BDO treatment as compared to vehicle treatment in *Ppara^HEPKO^* mice (Figure 6A). Cardiac triglyceride levels were also significantly decreased by 1,3-BDO treatment as compared to vehicle treatment in *Ppara^HEPKO^* mice (Figure 6B). Heart sections stained with PSR showed an excessive buildup of collagen in the cardiac tissue of *Ppara^HEPKO^* mice, which was attenuated by 6 weeks of 1,3-BDO treatment (Figure 6C). This result was confirmed by Western blotting of collagen I protein from the LV of *Ppara^HEPKO^* mice, which was significantly higher compared to *Ppara^FL/FL^* mice and significantly attenuated by 1,3-BDO treatment (Figure 6D).

### 2.7. Treatment with 1,3-BDO Lowers Natriuretic Peptides and Restores BDH1, Catalase, and AMPK, and Attenuates Markers of Apoptosis in the Ppara^HEPKO^ Mice

**Figure 7:**
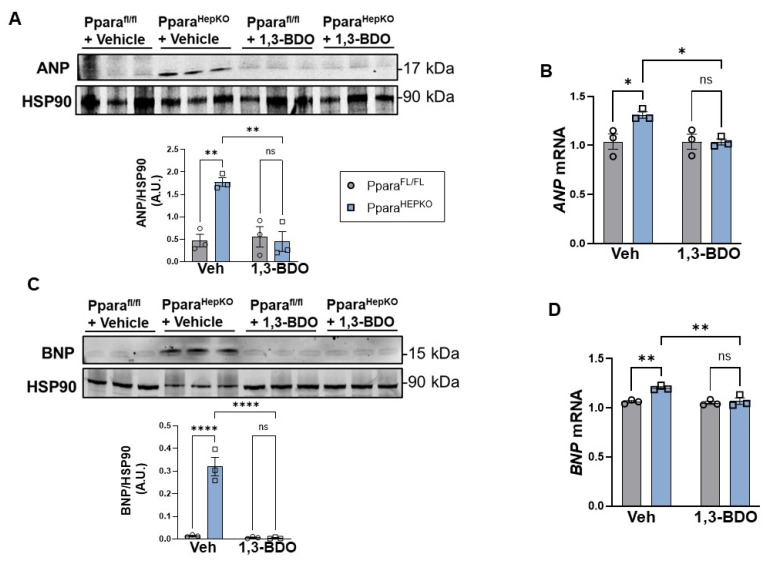
1,3-BDO treatment attenuates cardiac natriuretic peptides in *Ppara^HEPKO^* mice. (**A**) Western blot of atrial natriuretic peptide (ANP), (**B**) mRNA levels of cardiac ANP, (**C**) Western blot of B-type natriuretic peptide (BNP), (**D**) mRNA levels of cardiac BNP. Values are expressed as means ± SEM; * = *p* < 0.05, ** = *p* < 0.01, **** = *p* < 0.0001; *n* = 3/group. VEH, vehicle; 1,3-BDO, 1,3-Butanediol. Statistical analyses were performed using two-way ANOVA with genotype and treatment as factors, followed by Tukey post hoc test for all subsequent pair-wise comparisons. ns = not significant.

**Figure 8:**
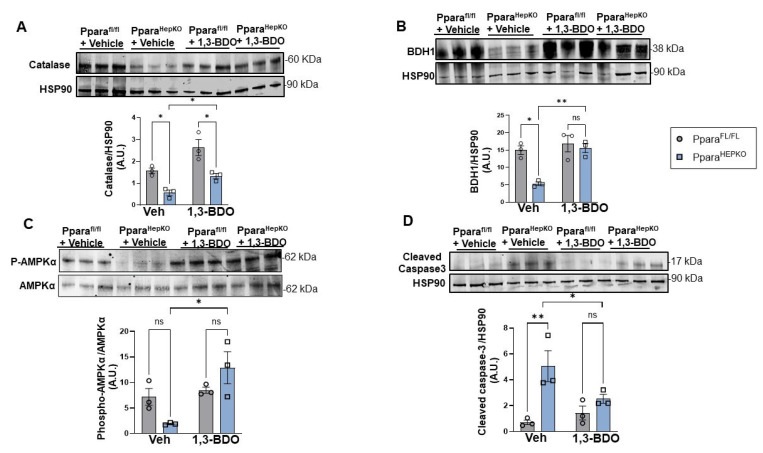
1,3-BDO treatment increased cardiac expressions of BDH1, catalase, and AMPKα in *Ppara^HEPKO^* mice. (**A**) Western blot of cardiac catalase, (**B**) Western blot of cardiac D-beta-hydroxybutyrate dehydrogenase (BDH1), (**C**) Western blot of cardiac phospho AMP-activated protein kinase alpha (AMPKα), (**D**) Western blot of cleaved caspase-3. Values are expressed as means ± SEM; * = *p* < 0.05, ** = *p* < 0.01; (**A**,**B**): *n* = 3/group. VEH, vehicle; 1,3-BDO, 1,3-Butanediol. Statistical analyses were performed using two-way ANOVA with genotype and treatment as factors, followed by Tukey post hoc test for all subsequent pair-wise comparisons. ns = not significant.

The levels of natriuretic peptides, ANP and BNP, were significantly increased in *Ppara^HEPKO^* mice as compared to *Ppara^FL/FL^* mice and were reduced by 1,3-BDO treatment in *Ppara^HEPKO^* mice (Figure 7A,B). Cardiac levels of BDH1, catalase, and pAMPK protein were attenuated in *Ppara^HEPKO^* mice as compared to *Ppara^FL/FL^* mice; however, treatment with 1,3-BDO for 6 weeks resulted in the restoration of these proteins in the hearts of *Ppara^HEPKO^* mice (Figure 8A–C). In addition, 1,3-BDO treatment attenuated cleaved caspase 3, a marker of apoptosis, in *Ppara^HEPKO^* mice (Figure 8D).

## 3. Discussion

CVD is the leading cause of death in patients with MASLD [15,16]. However, the mechanisms by which MASLD promotes CVD remain unknown. We have identified a model of MASLD in *Ppara^HEPKO^*. These mice exhibit many of the features of patients with “lean” MASLD, including hypertension, cardiac dysfunction, and increased vascular stiffness [6,7]. *Ppara^HEPKO^* mice exhibit decreased plasma levels of the ketone, β-hydroxybutyrate (BHOB) [17]. In the present study, we restored plasma BHOB levels by treating our mouse model of MASLD with the precursor compound, 1,3-butanediol (1,3-BDO), and examined the therapeutic effect of increased plasma BHOB levels in MASLD-induced CVD. BHOB is an important metabolite produced by the liver during ketogenesis, which occurs primarily under conditions of limited glucose availability, such as fasting and prolonged exercise [18]. Even though BHOB is synthesized mainly by the liver from fatty acids, it is not used by the liver due to a lack of the enzyme succinyl-CoA:3-oxoacid CoA-transferase (SCOT) that catabolizes the ketone into a usable form [19,20]. Instead, BHOB is transported in the bloodstream to extrahepatic tissues, including the heart, where it serves as an alternative fuel source, affecting cardiac function [19].

Restoring plasma BHOB levels significantly decreased blood pressure in *Ppara^HEPKO^* mice and did not affect blood pressure in *Ppara^FL/FL^* mice. This finding is significant because high blood pressure is common among patients with MASLD [21,22]. Several mechanisms by which increased BHOB levels could lower blood pressure in *Ppara^HEPKO^* mice are possible. BHOB has been shown to improve antioxidant defense in the kidney, which is associated with lower blood pressure in Dahl salt-sensitive rats on a high-salt diet [23]. BHOB increases the levels of endogenous antioxidants, such as catalase, which lowers blood pressure by mitigating the detrimental effects of hydrogen peroxide, a contributor to oxidative stress and the development of hypertension [23,24]. BHOB has also been shown to inhibit histone deacetylases (HDACs) [25,26]. HDAC inhibitors have been shown to reduce renal fibrosis and lower blood pressure in obesity-associated hypertension [27,28]. BHOB could also potentially lower blood pressure through both intrarenal and systemic vascular mechanisms. Vascular stiffness is a major driver of hypertension-related CVD, as it increases blood pressure and, in turn, impairs arterial wall function [29,30]. BHOB promotes vasodilation by activating potassium channels on endothelial cells [31]. In the present study, 1,3-BDO administration reduced vascular stiffness in PparaHEPKO mice; however, it is unclear whether this was a direct effect or attributable to the reduction in blood pressure, as potassium channel activity was not assessed. Further studies examining the potential role of the mechanisms described above are needed to clarify how increasing plasma BHOB levels lowers blood pressure and improves vascular stiffness in *Ppara^HEPKO^* mice.

1,3-BDO treatment improved indicators of systolic function, including stroke volume, cardiac output, ejection fraction, and end-diastolic volume in *Ppara^HEPKO^* mice. Similarly, 1,3-BDO treatment improved crucial indicators of left ventricular diastolic function, such as MV E, e’, the E/e’ ratio, and IVRT. Several studies have linked MASLD to the development of heart failure with preserved ejection fraction (HFpEF), in which the heart exhibits reduced relaxation [32,33]. The cardiac remodeling and systolic and diastolic dysfunction observed in *Ppara^HEPKO^* mice before restoration of BHOB levels may result from elevated blood pressure. Elevated blood pressure increases the heart’s afterload, thereby forcing the left ventricle to pump against greater vascular resistance to eject blood during systole [34]. To overcome this pressure overload, the left ventricular walls initially thicken as an adaptive response, allowing the heart to maintain cardiac output. However, the adaptive response of cardiac hypertrophy will eventually fail, transitioning to dilated cardiomyopathy and reduced cardiac efficiency [35]. In our previous study, we observed degenerative changes in the left ventricular anterior and posterior walls, resulting in thin left ventricular walls and structural changes that resemble those of dilated cardiomyopathy in *Ppara^HEPKO^* mice [9]. Dilated cardiomyopathy is a type of cardiac remodeling that is characterized by dilated left ventricles, thin and weakened left ventricular walls, impaired cardiac contraction, and reduced systolic function [36]. In the present study, 1,3-BDO treatment did not alter the left ventricular structure in *Ppara^HEPKO^* mice. The data from the present study indicate that increasing plasma BHOB levels improves cardiac function independent of reversing cardiac remodeling in *Ppara^HEPKO^* mice. One mechanism that may underlie the improved cardiac function in 1,3-BDO-treated *Ppara^HEPKO^* mice is the restoration of cardiac BDH1 and AMPK activity. Cardiac BDH1 is the rate-limiting enzyme in ketogenesis and ketolysis, and it is primarily involved in the utilization of ketone bodies as an energy source in the heart. It acts by converting BHOB into acetyl-CoA, which then enters the tricarboxylic acid (TCA) cycle to produce energy for the heart [18]. BDH1 expression is elevated in failing hearts to facilitate adequate ketone oxidation, thereby improving cardiac function and contractility [37]. This is in contrast to our present study, where we observed suppressed expression of cardiac BDH1 in *Ppara^HEPKO^* mice, which was restored by 1,3-BDO treatment. Furthermore, we observed that increasing plasma BHOB levels improves cardiac AMPK activity, which is suppressed in *Ppara^HEPKO^* mice. Cardiac AMPK activation maintains cardiac energy homeostasis. It is associated with improved systolic and diastolic function by enhancing energy metabolism, promoting mitochondrial health, reducing fibrosis, and restoring cellular repair mechanisms [38]. While the upstream signaling components and direct phosphorylation targets remain to be fully characterized, the possible molecular mechanism linking the loss of hepatic PPARα to the suppression of myocardial AMPK phosphorylation and BDH1 expression likely involves a lipotoxic signaling axis. The lipotoxicity associated with our MASLD model could be due to inhibition of Liver Kinase B1 (LKB1), the primary upstream kinase that phosphorylates AMPK at Thr172, while simultaneously upregulating protein phosphatases, such as PP2C, that deactivate AMPK [38,39]. Without functional LKB1, AMPK fails to become phosphorylated at Thr172. Active AMPK directly phosphorylates and co-activates PGC-1α, the master transcriptional co-activator driving the expression of PPARα [40,41]. Lacking this vital post-translational modification, PGC-1α cannot co-activate PPARα, thereby inhibiting downstream BDH1 Since AMPK directly phosphorylates PGC-1α, suppression of AMPK in vehicle-treated Ppara^HEPKO^ mice inhibits PGC-1α, which is responsible for the diminished cardiac BDH1 levels observed in our model. Exogenous restoration of BHOB via 1,3-BDO alleviates lipotoxic stress and might restore the LKB1/AMPK/PGC-1α/PPARα signaling axis that influences BDH1 expression. While our data demonstrate a clear association between suppressed hepatic PPARα signaling, reduced myocardial AMPK activation, and reduced BDH1 expression, in our future studies, we will target upstream kinases, such as LKB1 activity, to map the exact intracellular signaling driving these changes in the myocardium of the Ppara^HEPKO^ mice. Our data indicate that increasing exogenous BHOB levels may improve systolic and diastolic efficiency in our mouse model of MASLD by enhancing cardiac energy metabolism and contractility through the restoration of cardiac BDH1 expression and AMPK activity, even in the absence of reversed cardiac remodeling. However, this potential mechanism needs to be tested in future experiments.

One of the most interesting observations of the present study was the inability of 1,3-BDO treatment to have any significant impact on hepatic steatosis in *Ppara^HEPKO^* mice. Despite its failure to reduce hepatic steatosis, 1,3-BDO administration had a positive overall effect on the development of cardiovascular disease in this model. More interestingly, 1,3-BDO treatment reduced overall body weight gain and altered the distribution of body fat in *Ppara^HepKO^*, yet did not affect hepatic steatosis in this model. These results suggest that loss of hepatic PPARα can drive increases in hepatic steatosis independently of changes in body weight or adipose tissue distribution. They also demonstrate that improvements in cardiovascular health in patients with MASLD may be achievable without the correction of the underlying hepatic steatosis, which is believed to drive the disease. This finding has implications for the translational significance of these results as we seek treatments to prevent CVD in patients with MASLD. Although 1,3-BDO treatment did not affect hepatic lipids, it normalized alterations in plasma cholesterol levels in *Ppara^HEPKO^* mice. 1,3-BDO lowered the levels of total plasma cholesterol and LDL cholesterol, improved the LDL: HDL ratio, and also lowered cholesterol particles in the plasma. It is possible that these alterations in cholesterol and LDL cholesterol levels contributed to the beneficial actions of 1,3-BDO treatment, as hypercholesterolemia is a significant risk factor for CVD. In addition, high levels of LDL cholesterol are also associated with an increased risk of CVD. Interestingly, levels of ApoA1 were increased in *Ppara^HEPKO^* mice and decreased following 1,3-BDO treatment. The high levels of ApoA1 could potentially be a compensatory mechanism for the increased blood pressure and alterations in cardiac function observed in the *Ppara^HepKO^* mice and then lowered following 1,3-BDO treatment in response to improvements in these phenotypes. Further studies are needed to explore this possibility. 1,3-BDO treatment did not have a significant effect on plasma metabolites, such as amino acids, but decreased plasma levels of acetic acid and ethanol in *Ppara^HEPKO^* mice.

While increasing plasma BHOB levels with 1,3-BDO did not affect hepatic lipid levels in *Ppara^HEPKO^* mice, it significantly decreased lipid levels and reversed cardiac fibrosis. Cardiac fat accumulation is associated with an increased risk of CVD, which contributes to the primary cause of mortality in MASLD patients [42]. Administration of 1,3-BDO was highly effective in reducing cardiac fat accumulation in *Ppara^HEPKO^* mice, as further confirmed by the measurement of cardiac triglycerides. Cardiac lipid compartmentalization, which is concentrated in the interstitium, can occur during systemic metabolic dysregulation when the circulating lipid supply exceeds myocardial oxygen capacity [43,44]. There is a vicious cycle of apoptotic cardiomyocyte loss triggering cardiac fibrosis, and cardiac fibrosis in turn further induces cardiac apoptosis [45,46]. In addition, the accumulation of excessive collagen in the left ventricle of *Ppara^HEPKO^* mice disrupts the heart’s structural integrity and contributes to cardiac stiffness and impaired contractility. Recent studies have shown that increasing plasma BHOB levels can reverse cardiac apoptosis and fibrosis by attenuating oxidative stress and inflammatory pathways and by promoting macrophage polarization [46,47]. Future studies should assess the effects of 1,3-BDO treatment on these pathways in the hearts of *Ppara^HEPKO^* mice. Our data further demonstrate that increasing plasma BHOB levels ameliorates apoptosis-associated cardiac fibrosis in MASLD-induced CVD, as evidenced by a decrease in cleaved caspase 3, a marker of apoptosis, in *Ppara^HEPKO^* mice.

The functional significance of the changes in cardiac function resulting from 1,3-BDO treatment was assessed by subjecting the experimental animals to an exercise tolerance test. We observed increased running time until exhaustion and exercise performance in 1,3-BDO-treated *Ppara^HEPKO^* mice compared with untreated *Ppara^HEPKO^* mice. One potential mechanism for the increase in exercise performance is the attenuation of resting lactate levels by 1,3-BDO in *Ppara^HepKO^* mice, suggesting a shift from anaerobic to aerobic metabolism at rest. However, 1,3-BDO treatment did not significantly affect lactate levels after exercise. Under normal conditions, a healthy heart uses fatty acids (60–80%) as its primary fuel source because they require less oxygen to produce more energy than glucose [48]. Additionally, the heart relies to a lesser extent on other sources of energy, such as glucose (20–30%), ketones (10%), lactate, pyruvate, and amino acids (>5%) [37]. Because the heart has high energy demands, it exhibits metabolic flexibility, switching among various fuel sources in response to conditions such as fuel availability or disease [49]. Convincing evidence indicates that, in heart failure, in both human and animal models, there is a shift in fuel reliance from fatty acids toward increased utilization of ketone bodies [50,51,52,53,54]. The failing heart reprograms energy metabolism, increasing BHOB utilization from 10% to 30% [54]. This is due to a decrease in mitochondrial oxidative capacity, leading to an energy deficit in the heart [55]. Hence, the heart shifts toward increased ketone oxidation to compensate for reduced ATP production. In heart failure associated with obesity and diabetes, fatty acid oxidation increases; however, myocardial fatty acid oxidation decreases in heart failure associated with hypertension, leading the heart to rely on ketone oxidation [55].

We have consistently observed left ventricular dilation in *Ppara^HEPKO^* mice, resulting in increased end-diastolic volume, which suggestive of increased preload. Studies have shown that pathophysiological conditions with high preload are associated with elevated atrial natriuretic peptide (ANP) and brain natriuretic peptide (BNP), hormones produced by the cardiac muscle as a result of the heart’s compensatory response to increased volume overload [56,57]. Additionally, natriuretic peptides are recognized as important diagnostic and prognostic markers for heart failure [58]. In our present study, 1,3-BDO treatment attenuated the increases in both ANP and BNP in our MASLD-induced CVD model, which may be due to reduced end-diastolic volume, thereby reducing strain on cardiomyocytes. Studies have shown that, beyond cardiac energy metabolism, increases in plasma BHOB levels confer additional therapeutic benefits for the heart. These include body weight reduction, improved endothelial function, decreased blood pressure and cardiac remodeling, enhanced mitochondrial function, and reduced oxidative stress and inflammation [14,59,60,61]. Hence, various strategies to induce mild ketosis for the benefit of the heart could be employed over time; however, the exact mechanism by which they benefit the heart remains unclear. These strategies include the use of a ketogenic diet, the ingestion of ketone salts or ketone esters, and the ingestion of ketone precursors, such as 1,3-butanediol or medium-chain triglycerides [59]. Male mice were used in the current study because hepatic steatosis did not develop in females at the same time point as in male *Ppara^HEPKO^* mice. However, if females develop hepatic steatosis later in life to the same extent as males, future studies could test whether increased plasma BHOB levels attenuate CVD. Another limitation of the present study is the administration of the precursor molecule 1,3-BDO. 1,3-BDO is unlikely to be an ideal substrate for humans due to its extremely bitter taste. Other ketone precursors or a ketogenic diet may be needed to determine the beneficial effects of increased plasma BHOB in patients with human MASLD.

## 4. Materials and Methods

### 4.1. Mouse Models

All breeding, experimental procedures, and protocols of this study were performed in accordance with the NIH Guide for the Care and Use of Laboratory Animals and approved by the Institutional Animal Care and Use Committee of the University of Mississippi Medical Center. All animals were maintained in a temperature-controlled environment with a 12:12 h dark-light cycle at standard temperatures between 24 and 25 °C, with free access to food and water ad libitum. Studies were performed on 30-week-old male *Ppara^HEPKO^* and *Ppara^FL/FL^* mice since female mice do not exhibit significant MASLD-induced CVD at this age [9,61]. *Ppara^HEPKO^* and *Ppara^FL/FL^* mice were bred in our colony at the University of Mississippi Medical Center as previously described [17]. At 30 weeks old, they were randomly assigned to receive either 1,3 butanediol or vehicle treatment. A 20% concentration of 1,3 butanediol (1,3-BDO) (Thermo Fisher Scientific, Waltham, MA, USA), a precursor of BHOB, was administered in drinking water to the mice daily for 6 weeks. Regular drinking water was administered as vehicle control. All mice had free access to water and food throughout the experiment. Standard mouse chow consisted of 17% fat (Teklad diet #8604, Harland Laboratories, Inc., Indianapolis, IN, USA). Investigators were blinded to the genotypes of the mice at the time of experimentation. Mice were fasted for 8 h from early morning to afternoon before euthanasia via isoflurane overdose (5% inhaled, Isoflurane, Piramal Critical Care, Bethlehem, PA, USA), after which time blood and tissues were immediately collected and stored at −80 °C for further analysis.

### 4.2. Plasma BHOB Measurement

Plasma was collected for the measurement of BHOB at baseline before treatment and at 6 weeks after 1,3-BDO treatment. Blood was collected from mice after an 8 h fast via the retro-orbital sinus under inhaled isoflurane anesthesia (2% inhaled). Plasma BHOB was measured using a colorimetric assay kit from Cayman Chemical, Ann Arbor, MI, USA (Catalog No. 700190).

### 4.3. Blood Pressure and Heart Rate Measurements

Blood pressure and heart rate were determined by radiotelemetry. Telemetry catheters were placed in the left carotid artery under inhaled isoflurane anesthesia (2.5% in 1 L/min of O_2_) as previously described [9]. Mice were given buprenorphine-SR (Ethiqa-SR, Covetrus, Dublin, OH, USA, 1 mg/kg) as an analgesic agent 30 min before surgery and dosed after 72 h if necessary. Mice were allowed to recover for 7 days after surgery. Afterwards, continuous recording (sampling every 15 min for 10 s intervals) of systolic, diastolic, mean arterial pressure, and heart rate was collected for an additional 7 days. Data were collected and stored using the Dataquest ART data acquisition system (Data Sciences International, St. Paul, MN, USA), and Microsoft Excel was used for data analysis.

### 4.4. Echocardiography

Echocardiography was performed on mice after 6 weeks of 1,3-BDO treatment. A high-resolution echocardiography Vevo 3100 system with an MX400 probe (FUJIFILM Visual Sonics, Toronto, ON, Canada) was used to measure mouse cardiac function and structure. Initially, mice were anesthetized with 4% isoflurane in 1 L/min of O_2_, and maintained on 1–1.5% isoflurane when placed on the heated platform (39–40 °C) during measurements. Transthoracic echocardiographic measurements of cardiac output (CO), stroke volume (SV), ejection fraction (EF), left ventricular end-diastolic volume (LVEDV), and left ventricular mass were obtained from the parasternal long-axis view via the B-mode. These parameters are indicators of systolic function. Pulse-wave Doppler (PWD) imaging was used to measure the peak velocity flow in early diastole (E), peak velocity flow in late diastole (A), and isovolumetric relaxation time (IVRT), while tissue Doppler (TD) imaging was used to measure the early diastolic mitral annular velocity (e′) from the apical four-chamber view. The ratios of E/e′ and E/A were calculated and taken as indicators of diastolic function. Also, left ventricular dimensions, including left ventricular (LV) internal diameter during systole (LVID;s), LVID during diastole (LVID;d), LV anterior wall (LVAW) thickness, and LV posterior wall (LVPW) thickness during systole, were measured from the parasternal short-axis view via the M-mode. These parameters are indicators of LV structure. The imaging probe was also used to obtain a longitudinal view of the left carotid artery and abdominal aorta for the evaluation of vascular stiffness. Then the PWD was used to measure flow velocity in both vessels to obtain the pulsatility index (PI) and resistive index (RI).

All images from B-mode, M-mode, PWD, and TD were analyzed offline using the Vevo software (Vevo LAB Software; V. 3.2.6, Visual Sonics).

### 4.5. Exercise Tolerance Test

The exercise tolerance test was designed to match that previously reported by our laboratory [62]. Before the actual exercise tests, mice were placed on a six-lane rodent treadmill, approximately 10–15 cm wide and 92 cm long (Columbus Instruments, Columbus, OH, USA), and allowed to run for 5 min over 2 consecutive days to acclimate to the treadmill. During the actual measurements on the third day, mice were subjected to a graded maximal exercise test with the following settings: (speed, duration, grade)—(9 m/min, 2 min, 5 o), (12 m/min, 2 min, 10 o), (15 m/min, 2 min, 15 o), (18 m/min, 1 min, 15 o), (21 m/min, 1 min, 15 o), (23 m/min, 1 min, 15 o), (24 m/min, 1 min, 15 o) and (+1 m/min, each min thereafter, 15 o). A mild electric grid with an electric shock intensity of 0.4 mA was used to stimulate the mice to run. Mice were continuously monitored to immediately detect when they reached exhaustion. This point is defined as the time when animals maintain continuous contact with the shock grid for 5 s or touch the grid 5 times in 10 s or less. Resting blood lactate was measured 2 days before the exercise test via the tail vein using a lactate strip and meter (Nova Biomedical, Waltham, MA, USA), and lactate levels after exercise were also obtained immediately after mice were removed from the treadmill after running.

### 4.6. Hepatic and Cardiac Triglyceride Measurements

Triglyceride levels were measured in liver and heart samples using a colorimetric assay kit (Triglyceride Assay Kit, Abcam, Cambridge, UK), as previously described [9,17]. Briefly, tissues were weighed and homogenized in 1ml of 5% NP-40 solution. The homogenates were slowly heated to 100 °C and allowed to cool down to room temperature. Then, the homogenates were centrifuged, and the supernatants were diluted 10-fold before using the colorimetric assay kit as described by the manufacturer. Each standard, control, and sample from an individual mouse was run in duplicate and averaged to obtain group averages.

### 4.7. Analysis of Plasma Lipids and Metabolites

Plasma samples were obtained at the end of the experimental protocol, and plasma lipids and metabolites were measured by nuclear magnetic resonance (NMR) spectroscopy as previously detailed [17]. Experiments were performed using a 14.0 T Bruker magnet equipped with a Bruker AV-III console (Bruker, Billerica, MA, USA), and lipoprotein subclass analysis was performed using regression analysis of the NMR data as previously described [17].

### 4.8. Histology Staining

Furthermore, frozen sections of liver and left ventricle of the heart were stained with Oil Red O to determine lipid contents. Additionally, paraffin-embedded tissue sections were stained with a picrosirius red solution to determine the collagen content. The Mantra 2 Quantitative Pathology Imaging microscope, equipped with ×40 magnification, was used to capture images of Oil Red O staining and PSR levels, respectively. The Oil Red O and PSR stain percentages from four individual fields of view from four different sections were then determined using NIH ImageJ software (Version 3). Measurements from sixteen different sections per individual animal were averaged to obtain group averages.

### 4.9. Western Blot

Left ventricles were harvested, and protein concentrations were determined using the Bradford method. Expression of cardiac collagen I and III, atrial natriuretic peptide (ANP), B-type natriuretic peptide (BNP), 3-hydroxybutyrate dehydrogenase 1 (BDH1), catalase, and phospho AMP-activated protein kinase alpha (phospho-AMPKα) was determined by Western blot analysis as previously described [62]. Briefly, about 30 µg of each sample was separated by electrophoresis on a 10% SDS-polyacrylamide NuPAGE gradient gel (Invitrogen, Waltham, MA, USA) and transferred to a PVDF membrane. Membranes were incubated overnight at 4 °C with the following concentrations of primary antibodies: collagen I (1:2000, Abcam, AB21286), collagen III (1:1000, Santa Cruz Biotecnology (Dallas, TX, USA), sc271249), ANP (1:1000, Invitrogen, PA5-29559), BNP (1:1000, Invitrogen, PA5-96084), BDH1 (1:2000, Proteintech (Rosemont, IL, USA), 15417-1-AP), catalase (1:2000, Abcam, EPR20198), cleaved caspase-3 (1:1000, Cell Signaling (Danvers, MA, USA), D175), and phospho-AMPKα (1:1000, Cell Signaling, T172). Heat shock protein 90 (1:5000, Santa Cruz Biotechnology, sc-13119) was used as a housekeeping protein. After three washes in TBS  +  0.1% Tween 20, the membranes were incubated with secondary antibodies, such as donkey anti-rabbit (IRDye 800 LI-COR Biosciences (Lincoln, NE, USA) 926-32214) or goat anti-mouse (IRDye 680, LI-COR Biosciences (Lincoln, NE, USA), 926-68020) secondary antibodies (LI-COR Biosciences) (1:2000 dilution in TBS) for 2 h at 4 °C. Immunoreactivity was visualized and quantified by infrared scanning in the Odyssey system (LI-COR Biosciences, Lincoln, NE, USA).

### 4.10. RNA Extraction and PCR Analysis

Total RNA was harvested from vehicle and BHOB-treated *Ppara*^fl/fl^ and *Ppara*^HepKO^ mice by lysing hearts using RNeasy Mini Kits (QIAGEN Inc., Austin, TX, USA). Total RNA was read on a NanoDrop 2000 spectrophotometer (Thermo Fisher Scientific, Wilmington, DE, USA). Complementary deoxyribonucleic acid (cDNA) was synthesized using a high-capacity cDNA reverse transcription kit (Applied Biosystems, Waltham, MA, USA). Polymerase chain reaction (PCR) amplification of the cDNA was performed by quantitative real-time PCR using TrueAmp SYBR Green qPCR SuperMix (Alkali Scientific, Fort Lauderdale, FL, USA) with gene-specific primers (**ANP**: Forward-TACAGTGCGGTGTCCAACACAG/Reverse-TGCTTCCTCAGTCTGCTCACTC; **BNP**: Forward-TCCTAGCCAGTCTCCAGAGCAA/Reverse-GGTCCTTCAAGAGCTGTCTCTG; Eurofins Genomics LLC., Louisville, KY, USA). The thermocycling protocol used for this experiment has been previously described by our laboratory [17]. GAPDH was used as a reference gene for normalization.

### 4.11. Statistics

Statistical analyses were performed with Prism 10 (GraphPad Software, San Diego, CA, USA) using a two-way ANOVA with genotype and treatment as factors, followed by a Tukey post hoc test for all subsequent pair-wise comparisons. Results are expressed as mean ± SEM. Statistically significant differences were accepted at *p* values of 0.05 or less.

## 5. Conclusions

The current model of MASLD exhibits cardiovascular phenotypes highly similar to those observed in patients with human MASLD. Given the ever-growing epidemic of MASLD, it is critical to find therapies that can mitigate or reverse CVD in these patients, as this is the primary source of mortality and morbidity among them. The present study demonstrates that CVD can be effectively managed in these patients without correcting the underlying hepatic steatosis by restoring plasma BHOB levels through the administration of ketone precursors.

## Figures and Tables

**Table 1:** Organ and Adipose Tissue Weights. All organs were weighed at the end of the experimental protocol. HW/BW, heart weight to body weight ratio. KW/BW, kidney weight to body weight ratio. LW/BW, liver weight to body weight ratio. EFW, epididymal fat weight. VFW, visceral fat weight. RFW, retroperitoneal fat weight. * = *p* < 0.05. *Ppara^FL/FL^* + Veh, *n* = 14, *Ppara^HEPKO^* + Veh, *n* = 12, *Ppara^FL/FL^* + 1,3-BDO, *n* = 9, *Ppara^HEPKO^* + 1,3-BDO, *n* = 8. Statistical analyses were performed using two-way ANOVA with genotype and treatment as factors, followed by Tukey post hoc test for all subsequent pair-wise comparisons.

Parameter	*Ppara^FL/FL^* + Veh	*Ppara^HEPKO^* + Veh	*Ppara^FL/FL^* + 1,3-BDO	*Ppara^HEPKO^* + 1,3-BDO	Treatment *p* Value	Genotype *p* Value	Interaction *p* Value
Body Weight (g)	32.8 ± 0.9	31.6 ± 1.1	29.0 ± 1.3	29.3 ± 1.1	0.0074 *	0.6593	0.53
HW/BW (mg/g)	4.4 ± 0.2	4.6 ± 0.2	4.5 ± 0.1	4.6 ± 0.2	0.6354	0.5457	0.7583
KW/BW (mg/g)	11 ± 0.4	11.7 ± 0.4	12.2 ± 0.6	13.4 ± 0.8	0.0092 *	0.0864	0.5694
LW/BW (g/g)	0.04 ± 0.001	0.04 ± 0.001	0.04 ± 0.001	0.04 ± 0.001	0.4077	0.2014	0.8372
EFW (g)	0.57 ± 0.05	0.75 ± 0.08	0.5 ± 0.04	0.59 ± 0.08	0.0741	0.0397 *	0.4815
VFW (g)	0.25 ± 0.04	0.36 ± 0.07	0.23 ± 0.04	0.29 ± 0.03	0.3538	0.0893	0.5331
RFW (g)	0.25 ± 0.04	0.31 ± 0.05	0.22 ± 0.06	0.26 ± 0.04	0.5781	0.1936	0.7136
Total Fat (g)	1.35 ± 0.1	1.74 ± 0.2	1.2 ± 0.2	1.44 ± 0.2	0.1	0.0327 *	0.4635

**Table 2:** Plasma lipids and metabolites. Plasma samples were obtained at the end of the experimental protocol. Plasma lipids and metabolites were measured by NMR spectroscopy, as detailed in the methods. * = *p* < 0.05, *n* = 6 all groups. Statistical analyses were performed using two-way ANOVA with genotype and treatment as factors, followed by Tukey post hoc test for all subsequent pair-wise comparisons.

Parameter	*Ppara^FL/FL^* + Veh	*Ppara^HEPKO^* + Veh	*Ppara^FL/FL^* + 1,3-BDO	*Ppara^HEPKO^* + 1,3-BDO	Treatment *p* Value	Genotype *p* Value	Interaction *p* Value
Plasma Triglycerides (mg/dL)	47.2 ± 4.4	39.9 ± 2.8	45.1 ± 3.6	39.7 ± 1.9	0.723	0.069	0.771
Plasma Cholesterol (mg/dL)	55.8 ± 4	99.9 ± 8	58.2 ± 3.6	53.4 ± 3.6	0.0001 *	0.0004 *	0.0001 *
LDL Cholesterol (mg/dL)	19.5 ± 1.4	41.1 ± 4.4	20.5 ± 1.8	15.3 ± 1.5	0.0001 *	0.0053 *	0.0001 *
HDL Cholesterol (mg/dL)	44 ± 2.8	58.1 ± 6.4	46.7 ± 2.5	43.7 ± 3.7	0.2	0.1799	0.056
LDL:HDL Ratio	0.45 ± 0.03	0.56 ± 0.06	0.43 ± 0.02	0.33 ± 0.03	0.009 *	0.88	0.017 *
ApoA1 (mg/dL)	74.5 ± 3.4	100.1 ± 3.7	76.4 ± 2.9	70.4 ± 4.5	0.001 *	0.015 *	0.004 *
ApoA2 (mg/dL)	27.3 ± 0.5	24.8 ± 1.3	32.5 ± 1.6	24.3 ± 2.5	0.171	0.004 *	0.096
ApoB100 (mg/dL)	29.5 ± 1.5	33.1 ± 3.1	29 ± 0.8	25.8 ± 1.4	0.058	0.917	0.093
Total Cholesterol Particle Number (mmol/L)	536 ± 28	620 ± 52	528 ± 16	469 ± 26	0.026 *	0.72	0.045 *
VLDL Particle Number (mmol/L)	58.3 ± 8.1	45.1 ± 0.6	60.2 ± 8.1	49.1 ± 4.8	0.636	0.065	0.886
LDL Particle Number (mmol/L)	426 ± 15	624 ± 36	423 ± 14	390 ± 18	0.0001 *	0.0018 *	0.0001 *
Citric Acid (mmol/L)	0.15 ± 0.01	0.15 ± 0.01	0.13 ± 0.004	0.11 ± 0.008	0.008 *	0.243	0.402
Ethanol (mmol/L)	3.9 ± 0.2	4.2 ± 0.3	3.7 ± 0.2	3.3 ± 0.3	0.038 *	0.611	0.2

## Data Availability

The data underlying this article will be shared on reasonable request to the corresponding author.

## Abbreviations

The following abbreviations are used in this manuscript:

BHOB	Beta hydroxybutyrate
CVD	Cardiovascular Disease
PPARα	peroxisome proliferator-activated receptor α
MASLD	Metabolic-Associated Steatotic Liver Disease
1,3-BDO	1,3 butanediol
